# First observation of direct methane emission to the atmosphere from the subglacial domain of the Greenland Ice Sheet

**DOI:** 10.1038/s41598-018-35054-7

**Published:** 2018-11-09

**Authors:** Jesper Riis Christiansen, Christian Juncher Jørgensen

**Affiliations:** 1 Department of Geoscience and Natural Resource Management, University of Copenhagen, DK-1958 Frederiksberg C, Denmark; 2 Department of Bioscience, Aarhus University, DK-4000 Roskilde, Denmark

## Abstract

During a 2016 field expedition to the West Greenland Ice Sheet, a striking observation of significantly elevated CH_4_ concentrations of up to 15 times the background atmospheric concentration were measured directly in the air expelled with meltwater at a subglacial discharge point from the Greenland Ice Sheet. The range of hourly subglacial CH_4_ flux rate through the discharge point was estimated to be 3.1 to 134 g CH_4_ hr^−1^. These measurements are the first observations of direct emissions of CH_4_ from the subglacial environment under the Greenlandic Ice Sheet to the atmosphere and indicate a novel emission pathway of CH_4_ that is currently a non-quantified component of the Arctic CH_4_ budget.

## Introduction

Permafrost and glaciers have been hypothesized to function as important caps of methane (CH_4_) – a greenhouse gas 25–30 times more powerful than carbon dioxide (CO_2_)^1^ due to its higher energy absorption properties. Disintegration of these cryospheric caps could lead to large increases in CH_4_ emissions in the Arctic with a significant feedback to the global climate system^2,3^. Direct evidence of the occurrence, magnitude and temporal extent of cryospheric CH_4_ emissions are currently derived from Arctic wetlands^4^, lakes^5,6^ and sub-marine CH_4_ sources^7^. Subglacial sediments and glacial meltwater have been shown to hold the potential for CH_4_ production and emission^8–11^, due to anaerobic decomposition of organic carbon^2,10,12^ by methanogenic archaea. This subglacial CH_4_ may subsequently be oxidized by methanotrophs facilitated by atmospheric oxygen (O_2_) or O_2_ release from basal ice melting^11^. Reservoirs of CH_4_ hydrates have been found beneath ice sheets^1,13–15^ of which the future stability may change due to accelerated melting and marginal thinning of the ice sheet leading to potential emissions of CH_4_ to the atmosphere. These studies suggest that subglacial environments could be active components of the Earth’s CH_4_ cycle. However, observations of direct emissions of subglacial CH_4_ have so far not been documented and the importance of subglacial CH_4_ for cryospheric CH_4_ release in the Arctic thus remains unknown.

## Results

**Figure 1:**
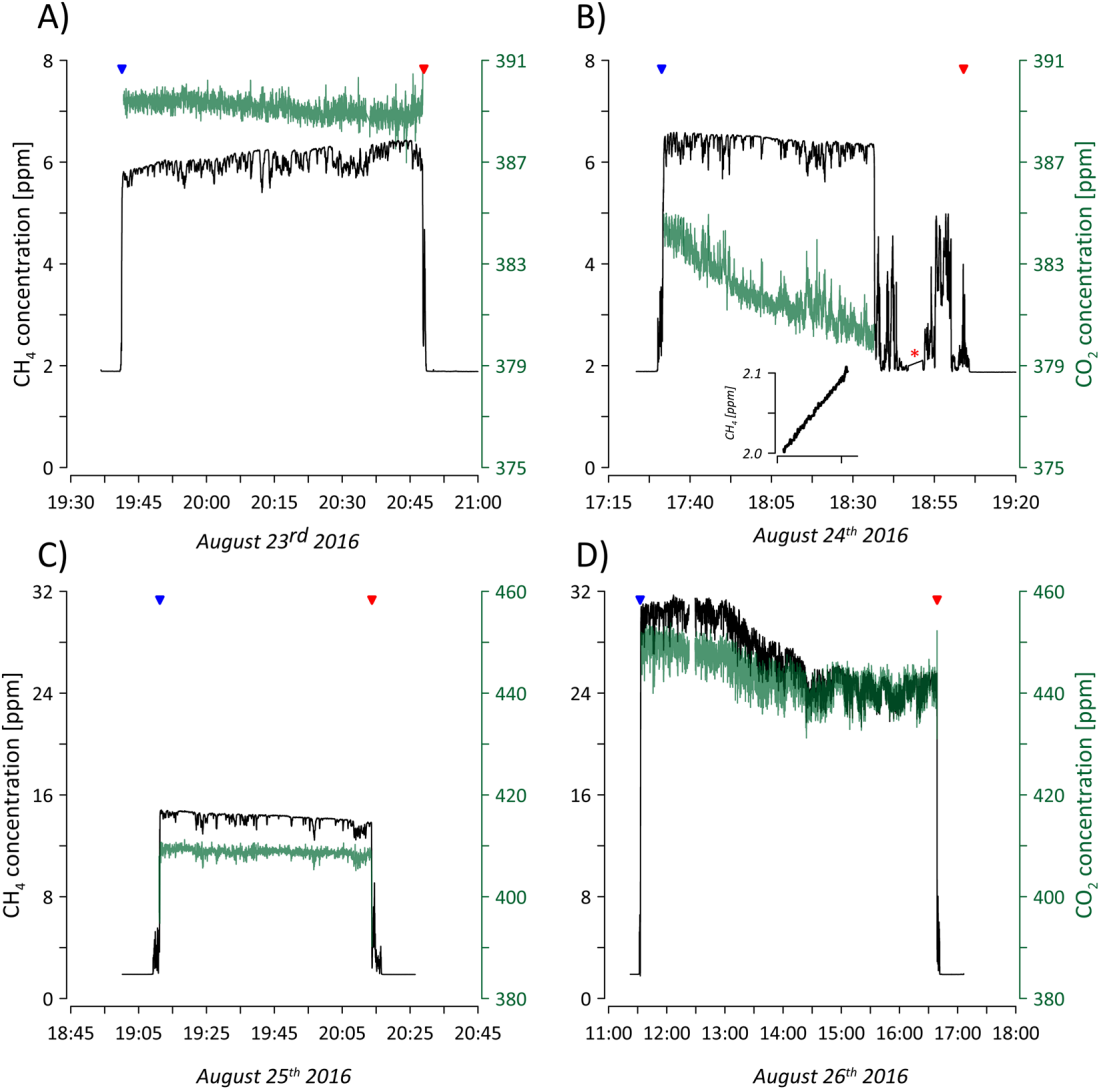
Measurements of CH_4_ (black) and CO_2_ (green) concentrations in subglacial air expelled from one lateral subglacial meltwater discharge point at the Isunnguata Sermia glacier, West Greenland on (**A**) August 23^rd^, (**B**) August 24^th^, (**C**) August 25^th^ and (**D**) August 26^th^ 2016. Blue and red triangles mark the start and end of CH_4_ and CO_2_ measurements in the subglacial cave, respectively. For (**B**) is also shown timeseries of CH_4_ concentrations measured in the air flowing through cracks in the ice next to the main lateral outlet. (see Fig. 3C for details). The red asterisk in (**B**) represent a closed chamber measurement (Fig. 3B) of the CH_4_ exchange between meltwater and the atmosphere (insert graph). The CO_2_ concentrations in the air is not shown due to contamination with human breadth resulting in highly fluctuating measurements of CO_2_ in the air outside the cave. This was not the case for CH_4_.

**Figure 2:**
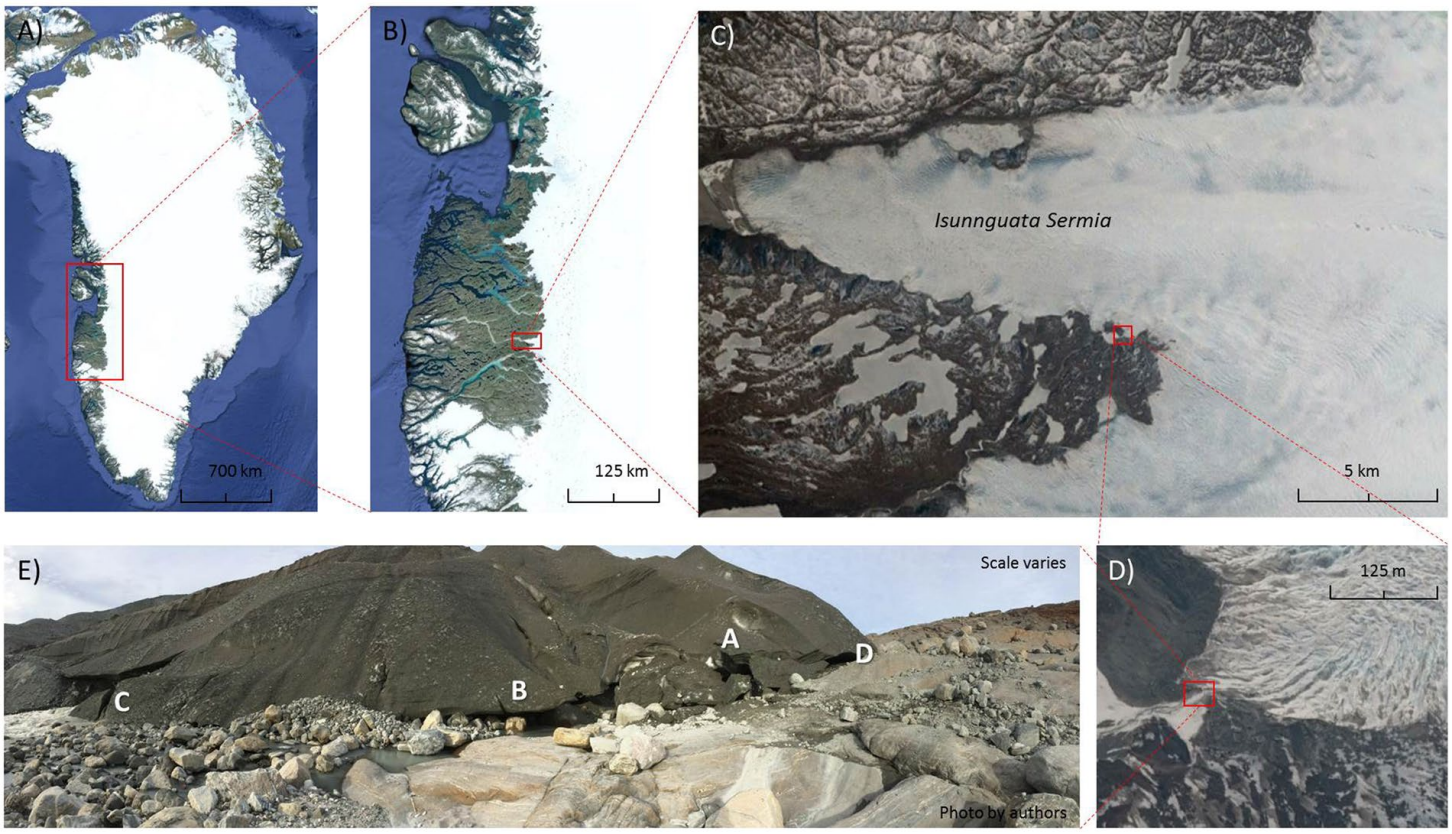
Location and detailed view of study site in Western Greenland. Source of satellite images: Google Earth (accessed 05–05–2017), Google Inc. 2017. Attribution to map providers: (**A**) IBCAO, U.S. Geological Survey, Landsat/Copernicus, (**B**) IBCAO, U.S. Geological Survey, (**C**) DigitalGlobe 2017, U.S. Geological Survey, (**D**) DigitalGlobe 2017. (**E**) Panoramic view of the margin of the Isunnguata Sermia Glacier. Letters A to D refer to the location of the four different modes of CH_4_ and CO_2_ exchange performed at the site (see Fig. 3 for details).

In the period between the 23^rd^ and 27^th^ of August 2016, CH_4_ and CO_2_ concentrations (Fig. 1A–D) were periodically measured in the air streaming out of one subglacial cavity connected to a lateral subglacial discharge point on the southern flank of the Isunnguata Sermia Glacier on the western Greenland Ice Sheet (Fig. 2). The measured subglacial CH_4_ concentrations were consistently higher than the atmospheric background concentration (at 1.9 ppm) and varied between 5.4 and 31.7 ppm (Fig. 1A–D). Subglacial CO_2_ concentrations varied around the atmospheric CO_2_ level (391 ppm) from 380 to 450 ppm. CO_2_ concentrations above the ambient level occurred with the highest CH_4_ concentrations on August 25^th^ and 26^th^ (Fig. 1C,D). High-frequency fluctuations in the subglacial air stream CH_4_ (range 0.12–0.75 ppm) and CO_2_ (range 0.21–1.49 ppm) concentrations were observed (Fig. 1A–D) during the measurements in the subglacial cave and the magnitude of the fluctuations increased with the subglacial CH_4_ and CO_2_ concentrations (Fig. 1A–D). The fluctuations were higher (except for CO_2_ on August 23^rd^ and 24^th^) than the natural fluctuations of the atmospheric background level CH_4_ and CO_2_ measured on site with a portable CH_4_ and CO_2_ analyzer (see Materials and Methods). The fluctuations indicate that the subglacial CH_4_ and CO_2_ is mixed by air with lower concentrations, which likely is atmospheric air entering the subglacial system via surface moulins or other pathways which connects the subglacial system to the atmosphere. A net emission of CH_4_ from the subglacial meltwater to atmosphere was also measured directly (Fig. 1B) showing that the meltwater itself can be a direct source of CH_4_ and sink of CO_2_. In the free air immediately above the subglacial meltwater outside the subglacial cavity, CH_4_ concentrations fluctuated rapidly from the ambient atmospheric concentration to approximately 5 ppm CH_4_ (Fig. 1B). There was no exchange of either CH_4_ and CO_2_ (not shown) from the sediment at the edge of the glacier (Fig. 1D).

Based on field observations (Supplementary Video Material) and wind tunnel experiments (Supplementary Fig. S1), the velocity of the subglacial air stream was estimated to fall in the range of 0.2 m s^−1^ to 2 m s^−1^ (Supplementary Fig. S2). From field observations, the cross-sectional area of the subglacial cavity where air was streaming out was estimated to be in the range of 1–2 m^2^. Using a cross sectional area of 1 m^2^, the estimated range in air velocity of 0.2–2 m s^−1^ and the span of measured CH_4_ and CO_2_ concentrations, the total hourly cumulative flux rate per square-meter cross-section area were estimated to range from 3.1 to 134 g CH_4_ m^−2^ h^−1^ and 537 to 6360 g CO_2_ m^−2^ h^−1^ (Table 1). However, it should be emphasized that these flux estimates represent the total emission of CH_4_ and CO_2_ from a so far unknown subglacial catchment area and the average emission per square meter catchment remains unknown.

**Table 1:** Flux rates of subglacial CH_4_ and CO_2_ to the atmosphere.

Air velocity (m s^−1^)	CH_4_ (ppm)		CO_2_ (ppm)	
	*5*.*4*	*31*.*7*	*380*	*450*
	*(minimum)*	*(maximum)*	*(minimum)*	*(maximum)*
	g CH_4_ m^−2^ h^−1^	g CH_4_ m^−2^ h^−1^	g CO_2_ m^−2^ h^−1^	g CO_2_ m^−2^ h^−1^
*0*.*2–2*.*0*	**3**.**1***–***31**	**13** *–* **134**	**537** *–* **5370**	**636** *–* **6360**

Estimates of measured cumulative hourly flux rates of subglacial CH_4_ (g CH_4_ m^−2^ h^−1^) and CO_2_ (g CO_2_ m^−2^ h^−1^) under minimum and maximum observed *in situ* concentrations and plausible range of air velocities of the subglacial air stream. Area unit signifies cross-sectional area of subglacial cave.

Acknowledging the fact that any conclusions on the overall climatic implication from this type of CH_4_ release would be premature due to the limited spatiotemporal observation of the phenomena, the presented measurements are to our knowledge the first showing direct emissions of CH_4_ and CO_2_ in the air being ventilated from the subglacial environment below the Greenland Ice Sheet to the atmosphere. The only previous reported study which similarly measured CH_4_ concentrations in the air mass directly at the glacial edge of the Greenland Ice Sheet was done in a dry subglacial cave in the Kangerlussuaq area at the Greenland Ice Sheet^16^. In this dry setting, CH_4_ concentrations were similar to the ambient atmospheric background level of CH_4_ at nearby field sites and the natural atmospheric background measured at the summit station on the Greenland Ice Sheet^17^.

## Discussion

The highly elevated CH_4_ concentrations and calculated flux rates of this study show an apparent correlation with a moving air mass induced by flowing subglacial meltwater. The observation of direct emissions of subglacial CH_4_ to the atmosphere supports earlier findings in the area, namely that this region of the Greenland Ice sheet may be a source of CH_4_ to the atmosphere^8^. The direct emission of gaseous CH_4_ also shows that biologic aerobic oxidation of CH_4_ in the subglacial domain may not be able to fully mitigate subglacial CH_4_ production^11^. Nonetheless, the process of CH_4_ production and location of the source of the measured subglacial CH_4_ is still unknown. Possible explanations for the presence of subglacial CH_4_ are either release of old (i.e. radiocarbon dead CH_4_) microbial and/or thermogenic gas from the subglacial domain^1^ which could be formed and stored under the Ice Sheet in the presence of a gas-hydrate stability zone^13,15^. Following entrainment in the subglacial water this old CH_4_ could be released following degassing of saturated subglacial meltwater in a similar process as described in Dieser *et al*.^8^. Alternatively, the emitted CH_4_ could be a product of more recently *in situ* produced biological CH_4_ from subglacial sediments^10,18^ or intermediately aged CH_4_ formed in carbon containing sediments capped during neoglacial readvances^3^. Finally, the possibility exist that the emitted CH_4_ could be a combined product of mentioned sources, which could be transported to the emission point via either running meltwater at the bedrock-glacier bed interface or as part of a deeper groundwater aquifer below the Isunnguata Sermia Glacier^19^. A plausible mechanism for the observed CO_2_ sink is the dissolution of CO_2_ in undersaturated meltwater resulting in a net uptake of atmospheric CO_2_^20^.

Future research efforts could be guided by two hypotheses focusing on the interrelations between meltwater volumes and glaciological dynamics. Thus, the release of subglacial CH_4_ and CO_2_ could be (1) caused by venting of the subglacial drainage system if atmospheric air entered the subglacial system due to alternating surface meltwater volumes. This could push out any gas that had accumulated in non-deformed englacial cavities through to the subglacial drainage system resulting in a short-lived but intense period of emissions. In this context, the glacial deformation of the meltwater drainage system will likely determine releases of gases to the atmosphere. Alternatively, (2) continuous emissions extending over the entire melt season could be due to a constant degassing of subglacial meltwater at the margin. This implies also that the subglacial emission would be tightly linked to the meltwater volume and the coupling between climate and supraglacial melting. Finally, obtaining data on the composition of C-isotopes in the emitted CH_4_ and CO_2_ will shed light on the origin of the emitted C-gases.

Despite these uncertainties in the origin of subglacial CH_4_ and CO_2,_ the mechanism of release and the limited extend of the measurement period, our measurements present the first evidence of a new pathway for direct interaction between the subglacial carbon cycle below the Greenland Ice Sheet and the atmosphere through the direct emission of CH_4_ and CO_2_. However, caution should be taken before drawing unjustified conclusions about the importance of subglacial CH_4_ emission in the Arctic CH_4_ budget and potential climate impact. Improving our understanding of the overall importance of subglacial CH_4_ emissions for the Arctic CH_4_ budget is therefore to expand the documentation of the spatial and temporal occurrences of subglacial emissions along the Greenland Ice Sheet. Also, further investigation into the release mechanism from the source area(s) can help resolve whether expected future increases in meltwater runoff due to increased surface melting and thinning of the ice sheet in a warming climate will affect the magnitude of subglacial CH_4_ emissions.

## Materials and Methods

### Study site

The study site is located at a lateral subglacial meltwater discharge point on the southern flank at the terminus of the Isunnguata Sermia Glacier at the western margin of the Greenland Ice sheet (67°09′16.40′′N 50°04′08.48′′W) (Fig. 2A–D). The site was at an elevation of 450 meter above sea level. The lateral outlet has been the focus of previous investigations of the geochemical composition of subglacial meltwater^21,22^ as well as drilling projects to assess groundwater chemistry below the Isunnguata Sermia Glacier^23^.

**Figure 3:**
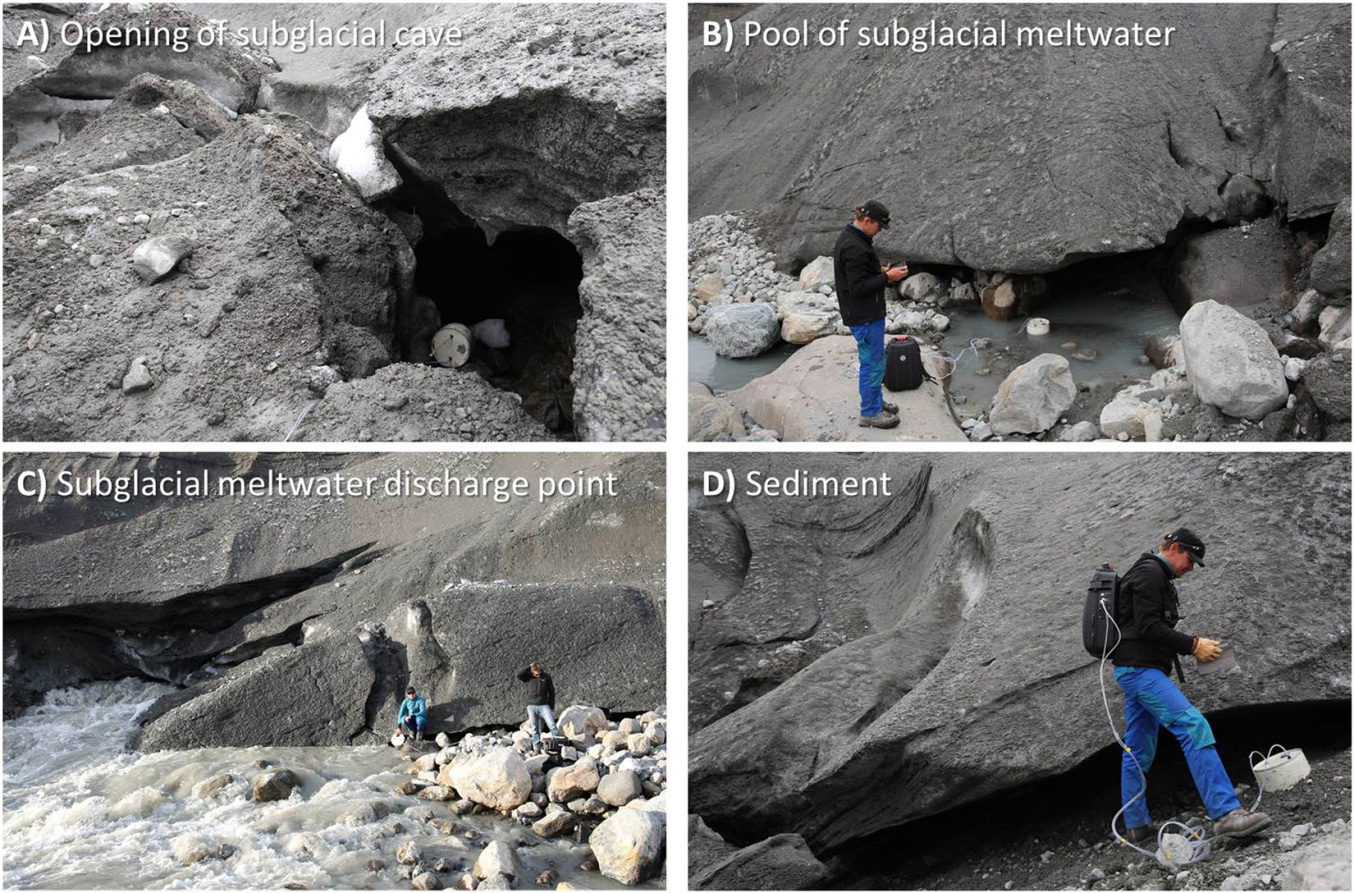
Modes of CH_4_ and CO_2_ measurements at the subglacial discharge point. (**A**) Open chamber inside the subglacial cave, (**B**) Closed chamber placed in a pool of subglacial meltwater, (**C**) open chamber placed in front of an ice crack connected to the subglacial discharge point and (**D**) Closed chamber placed on sediment beneath the ice edge. For (**B**,**D**) the chamber was connected to the analyzer forming a closed recirculation system for the measurement of the meltwater/sediment atmosphere exchange of gases, respectively. The mobile CH_4_ and CO_2_ analyzer (G4301, Picarro Inc.) can be seen in (**B**–**D**).

The area in front of the meltwater outlet consisted of the abraded granodioritic gneiss bedrock with large boulders and patches of gravel, sand and silt deposited by flowing meltwater (Fig. 2E). In close proximity to the subglacial meltwater outlet were air-filled cavities in the ice through which air constantly streamed from underneath the Ice Sheet to the atmosphere. Direct measurements of the subglacial CH_4_ and CO_2_ concentrations in the subglacial air streams from the air-filled ice cavities took place in the period August 23^rd^ to 26^th^ 2016. The 3-dimensional shape of the subglacial cavity was highly irregular, but the cross-section area (shown in Fig. 2E – marked by an A and Fig. 3A) at the terminus was estimated to be approximately 1–2 m^2^.

The landscape in the Kangerlussuaq area is typical of west Greenland, intersected by numerous long, narrow and up to 600 meter deep valleys in East to West direction. This type of topography extends below the ice sheet, in places reaching hundreds of meters below sea level. Groundwater recharge and discharge of the area is restricted to taliks, e.i. unfrozen zones in the permafrost, beneath large lakes, rivers and fiords^19^.

Deglaciation in the southern West Greenland area started around 12,300 years BP and the ice sheet margin reached its present position between 6,500 and 7,000 years BP. At approximately 6,000 years BP, the ice margin was east of its present position. Neoglacial re-advance may have started as early as 4,800 years BP and culminated about 2,000 years BP. During the the Little Ice Age (LIA) the ice sheet advanced again reaching a maximum position around 1850 AD with the maximum extent of the edge of the Isunnguata Sermia approximately 50–200 meters beyond its current position^19^.

Continuous permafrost extending to 350 meters below the surface has been reported at the study area^23^. The Isunnguata Sermia Glacier and the surrounding glaciers are underlain by granodioritic gneiss with no or only little sediment or organic material^21^. The glacier is warm based with an annual ice flow of 150–200 meters and surface meltwater reaches the base of the glacier^21^. The meltwater at the discharge point has been characterized as acidic (pH between 5.82–6.53)^22^ and poorly mineralized and may represent an outlier in terms of the pH of the subglacial meltwater^24^. However, the reported range in pH values of the subglacial meltwater with a seasonal average of 6.8 ± 0.5 at a nearby subglacial meltwater discharge point^8^ are within the range of typical pH values for silica-bedded glaciers and ice sheets (5.1 to 7.8)^24^. Borehole geochemistry at the site suggests that glacial meltwater extends deep in bedrock fractures below the Isunnguata Sermia Glacier and that groundwater below the permafrost is flowing away from the glacier margin^23^.

### *In situ* measurements of CH_4_ and CO_2_

*In situ* concentrations of CH_4_ and CO_2_ of the subglacial air were measured using a portable greenhouse gas analyzer based on state-of-the-art cavity ring-down spectroscopy with a measurement precision of 3 ppb and 0.4 ppm for CH_4_ and CO_2_, respectively and a sampling frequency of 1 hz (GasScouter G4301, Picarro Inc., CA, USA). The analyzer was connected to a flux chamber via a PVC tube (4 meter) which was deployed under the following ways: (1) the open chamber was lowered into the opening of one subglacial ice cavity (Fig. 3A), (2) the chamber was placed in a pool of subglacial meltwater forming a closed loop where the CH_4_ exchange between the meltwater and atmosphere was measured (Fig. 3B), (3) the open chamber was placed directly in front of cracks near the primary discharge point of the subglacial meltwater channel (Fig. 3C) and (4) the closed chamber was placed on top of loose sediments at the edge of the ice (Fig. 3D). Air temperature and relative humidity of the subglacial air stream was continuously measured inside the open chamber using a datalogger unit (TinyTag Ultra 2 – TGU-4500, Gemini Data Loggers Ltd, UK). The temperature and relative humidity of the subglacial air stream was constant at a level of 0.2 °C and 100%, respectively.

### Air flow velocity of the subglacial wind

The velocity of the air flow in the subglacial cavity was estimated by combining *in situ* observation of a smoke fan placed directly in the air filled cavity in the ice with a verification experiment in the laboratory using a small-scale wind tunnel with smoke injection. The general assumption was that movement of smoke when entrained in moving air in the ice cavity would be identical to movement of smoke entrained in moving air in a wind tunnel. The wind tunnel was constructed using a circular array of parallel straws to create laminar flow placed in the inlet end of a cardboard tube (inner diameter 10 cm). At the outlet end of the tube, a 12 V fan generated a laminar air flow that could be varied by controlling the power of the fan with an adjustable power supply. The flow velocity inside the tube was measured with a hot wire anemometer (model Testo 425, Testo SE & Co KGaA, Germany). Generated wind speeds in the tunnel ranged from 0.2 to 1.2 m s^−1^. Details on the wind tunnel setup and test is presented in Supplementary Material.

Video footage of *in situ* smoke movement in the air filled cavity in the ice was compared with observations of the smoke fans in the laboratory under wind speeds of 0.2, 0.5 and 1.2 m s^−1^ (Supplementary Video Material). This approach provided a best estimate of the *in situ* velocity of air flow in the subglacial cavity, and indicated that air speeds in the subglacial cavity reached at least 1.2 m s^−1^ and probably exceeded this value. We thus estimated that the likely range of air flow velocities in the subglacial cavity had a flow range between 0.2–2 m s^−1^ which was used for the estimation of the subglacial CH_4_ and CO_2_ flux rates.

### Flux rate calculation

Cumulative hourly flux rates of subglacial and CO_2_ (g m^−2^ h^−1^) were calculated combining a constant air flow rate through a 1 m^2^ cross-section area of the subglacial cavity and the measured CH_4_ and CO_2_ concentrations over a one hour measurement period (Equation 1). Flux rates are reported on an hourly basis as this was the maximum measurement time on August 23^rd^–25^th^. Concentrations were converted to mass using the ideal gas law.

1 $${F}_{C{O}_{2}/C{H}_{4}}=C\ast \bar{{\rm{\upsilon }}}\ast A\ast \frac{273.15}{({M}_{v}\ast (273.15+{T}_{a}))}\ast M\ast 3600\ast {10}^{-6}$$

where ${F}_{C{O}_{2}/C{H}_{4}}$ is the flux rate in g CH_4_/CO_2_ m^−2^ h^−1^, *C* is the measured 1 hz dry mole fraction concentration (µmol mol^−1^) of CO_2_ or CH_4_, *ῡ* is the wind speed (m s^−1^), *A* is the cross sectional area (m^2^), *M*_*v*_ is the molar volume (m^3^ mol^−1^), *T*_*a*_ is the air temperature (°C) measured in the cavity, *M* is the molar mass of CO_2_ or CH_4_ (g mol^−1^), the multiplier 3600 converts the flux to hourly values and the constant 10^*−6*^ converts the flux from µg to g CO_2_/CH_4_.

## Electronic supplementary material

Supplementary information

Supplementary video

## Data Availability

The dataset of CH_4_ and CO_2_ concentrations presented in Fig. 1 are available from the PANGAEA data repository (10.1594/PANGAEA.892391).
