# Supplementary material for: First observation of direct methane emission to the atmosphere from the subglacial domain of the Greenland Ice Sheet
Source: Sci Rep. 2018 Nov 9;8:16623. doi: 10.1038/s41598-018-35054-7 (PMC6226494; doi:10.1038/s41598-018-35054-7)
Supplement: Supplementary file 1 — Supplementary information [file 41598_2018_35054_MOESM1_ESM.docx]

**Supplementary material to First observation of direct methane emission to the atmosphere from the subglacial domain of the Greenland Ice Sheet**

By Jesper Riis Christiansen^1,*^ & Christian Juncher Jørgensen^1,2^

Author affiliations:

^1^Department of Geoscience and Natural Resource Management, University of Copenhagen, DK-1958 Frederiksberg C, Denmark

^2^ Department of Bioscience, Aarhus University, DK-4000, Roskilde, Denmark

*corresponding author – e-mail: jrc@ign.ku.dk

**Simulation of subglacial air flow velocity using a simple wind tunnel setup**

During the field measurements, a noticeable but non-quantified air flow was streaming to the atmosphere through the opening of the subglacial cave. As the flux rate of CH_4_ emission from the subglacial domain is directly proportional to the air flow velocity, we replicated the *in situ* observation where a smoke fan from a cigarette was placed in the subglacial, by constructing a simple wind tunnel (Figure S1) capable of generating air flow velocities between 0.2 to 1.2 m/s (Figure S2). The air flow velocity in the wind tunnel was generated using a standard 12 volt computer fan where the speed of the fan was varied using an adjustable voltmeter (Figure S2 - 1 & 2) with a linear voltage to air flow velocity relationship (Figure S3). The effective air flow velocity was measured directly in the air stream with a hot wire anemometer (Figure S2 – 3 & 4). Approximate laminar air flow in the wind tunnel was achieved using a circular array of straws at the inlet of the wind tunnel (Figure S2 – 5). When the fan was turned on, smoke was sucked through the array and the deformation of the smoke fan could be observed through a side window in the wind tunnel (Figure S2 – 6).

The degree of smoke deformation in the wind tunnel was visually compared to *in situ* video footage of a smoke fan placed in the subglacial air stream inside the subglacial cave (Supplementary video material). Based on the visual comparison, it was concluded that the air flow velocity in the subglacial cave likely exceeded the maximum wind speed (1.2 m/s) we could generate with the wind tunnel.


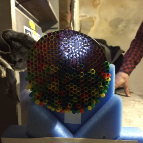

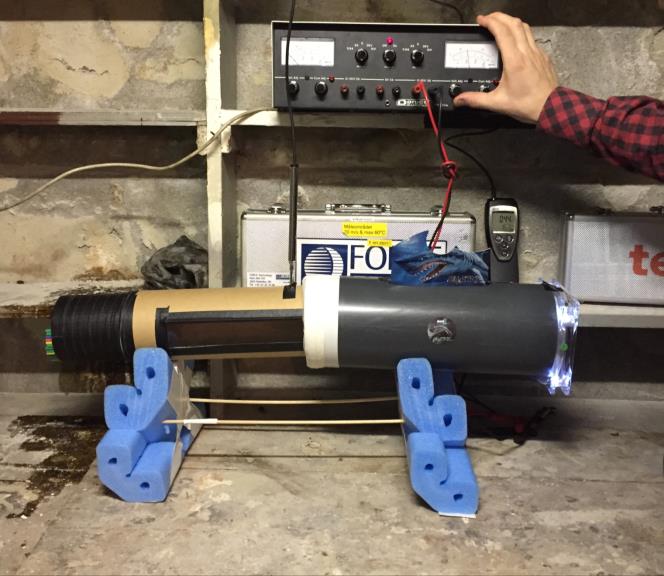


**4**

**2**

**5**

**6**

**3**

**1**

1. Adjustable volt meter

2. 12 volt computer fan

3. Hot wire anemometer

4. Reader for anemometer

5. Laminar flow array

6. Window for observation

**5**

Figure S1 Laboratory setup of the custom-built wind tunnel for simulation of *in situ* wind speed conditions in the subglacial cave. Numbers refer to legend in picture.


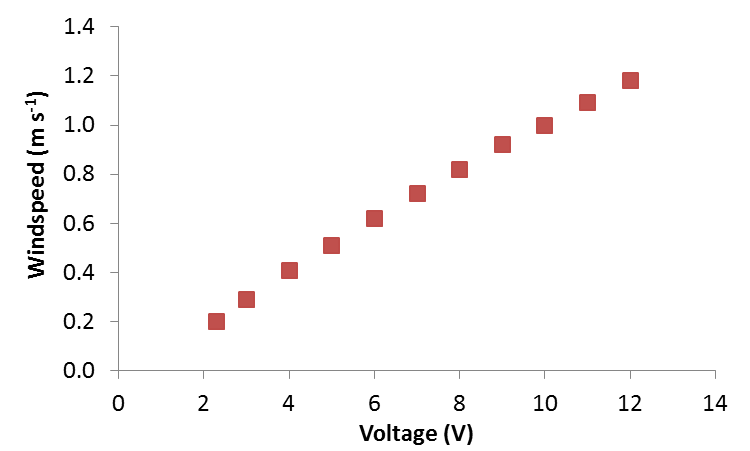


Figure S2 Relationship between voltage (V) and wind speed (m s^-1^) in the wind tunnel.

The operational range of the fan was between approximately 2 to 12 volts direct current, corresponding to a minimum air velocity of approximately 0.2 m s^-1^ and a maximum air velocity of 1.2 m s^-1^ (Figure S2).
